# How Do Mothers’ Parental Attributions Affect Child Outcomes from a Positive Parenting Intervention? A Mediation Study

**DOI:** 10.1007/s10578-019-00942-0

**Published:** 2019-11-15

**Authors:** Vilas Sawrikar, David J. Hawes, Caroline Moul, Mark R. Dadds

**Affiliations:** 1 University of Edinburgh, Edinburgh, UK; 2 University of Sydney, Sydney, NSW Australia

**Keywords:** Parent training, Parental attributions, Optimising outcomes, Parenting, Parent–child relationships

## Abstract

Problematic parental attributions refer to negative causal explanations for child problem behaviour and are known to predict parenting intervention outcomes. This study examines alternative accounts of how mothers’ problematic parental attributions, operationalised as negative pre-treatment and change resistant parental attributions during treatment, may affect child behaviour outcomes from a parenting intervention program. Putative mediators included parental feelings about the child and use of harsh discipline. Participants were 163 families with children aged from 3 to 16 referred to specialist clinics for the treatment of conduct problems. Measures were collected as part of pre-treatment, post-treatment, and 3-month follow-up assessments. Mothers’ pre-treatment and change resistant parental attributions were associated with smaller improvements in parental feelings at the end of treatment which in turn were associated with greater use of harsh discipline. Greater use of harsh discipline was associated with greater conduct problems overall. Smaller improvements in parental feelings mediated the effects of pre-treatment and change resistant parental attributions on outcomes in mothers’ use of harsh discipline and mediated the effects of change resistant parental attributions on outcomes in child conduct problems. Smaller improvements in parental feelings about the child may act as a mechanism that explains the impact of problematic parental attributions on treatment outcomes.

**Electronic supplementary material:**

The online version of this article (10.1007/s10578-019-00942-0) contains supplementary material, which is available to authorized users.

A wealth of research indicates that Behavioural Parent Training (BPT) is the most effective form of intervention for reducing child conduct problems [1, 2]. However, the impressive data demonstrating the efficacy of BPT is counterbalanced by evidence that approximately one-third to up to a half of treated families find it difficult to achieve and maintain the benefits of BPT [3, 4]. Researchers have subsequently suggested that personalising interventions by matching adjunctive components to family risk characteristics may provide a method for optimising treatment outcomes [5–7]. We recently showed that mothers’ parental attributions about the causes of child behaviour are a unique predictor of child outcomes [8]. This study extends the findings by examining potential mechanisms by which mothers’ parental attributions are linked to outcomes in BPT to better understand the role of parental attributions in BPT for conduct problems.

Research involving mothers of children with conduct problems shows that these parents are more likely to regard child negative behaviours as caused by factors that are internal, stable, and global to the child [9–11]. Further, families with mothers exhibiting these types of parental attributions prior to participating in BPT treatment are known to be at risk for poor child behaviour outcomes [8, 12]. This suggests that targeting mothers’ parental attributions within BPT may improve outcomes. However, recent findings indicated negative pre-treatment parental attributions were only prevalent in a small proportion of mothers attending BPT and were amenable to change during treatment [8]. Changes in parental attributions are hypothesised to occur experientially from parents benefitting from participation in BPT that may change their perceptions of their child [13]. However, a lack of positive progress in treatment may relate to change resistant parental attributions as an important unique predictor of poor child behaviour outcomes [8]. This suggests that best possible interventions might need to be tailored for the subset of families with mothers showing negative pre-treatment and change resistant parental attributions to optimise BPT outcomes.

The problem is very little research has been undertaken on how this might be done as part of clinical practice (see Sawrikar and Dadds [14] for review). Further, directly targeting parental attributions to achieve better child behaviour outcomes is not guaranteed to produce better outcomes beyond the standard program [15]. As such, Sawrikar and Dadds [14] suggests attention should be given to understanding the role of parental attributions in BPT for conduct problems by studying how parental attributions affect child behaviour outcomes. Consistent with frameworks establishing evidence-based standards for tailoring treatments, support for including components focusing on parental attributions is provided when there is evidence that it may affect target mechanisms underlying functional improvement [5, 16]. The current study evaluates whether this potentially is the case for targeting mothers’ parental attributions in BPT by investigating alternative accounts of how mothers’ parental attributions affect child behaviour outcomes.

We note that fathers also demonstrate problematic parental attributions that uniquely predict BPT outcomes [8]; however, inconsistent participation of fathers in post-treatment assessments in this study meant their data were excluded from analyses. Notwithstanding this limitation, substantial motivation exists for wanting to understand how mothers’ problematic parental attributions affect parenting intervention outcomes. Mothers generally demonstrate more problematic parental attributions for explaining child problem behaviours than fathers [8, 17–19], and more likely to attribute child conduct problems to factors external to themselves than fathers [20, 21]. Importantly, mothers have been shown to demonstrate change resistant parental attributions that predict poorer child behaviour outcomes after participation in BPT; a finding not replicated for fathers [8]. This research suggests that mothers are more likely to demonstrate problematic parental attributions of a prognostic nature in BPT.

For several decades, researchers have presented social cognitive models for proposing how parental attributions affect BPT outcomes. These models hypothesise that parents who view the cause of their child’s problems to be internal, stable, and controllable are less accepting of and engaged in parent training [13, 22, 23]. Child-internal causal attributions may mean that parents expect that the focus of treatment should be on the child and parents need to have little involvement in treatment [24]. This is problematic since retaining parents and having them attend regular treatment sessions are arguably basic necessities for maintaining the fidelity of BPT [25]. Despite these lines of argument, however, Sawrikar and Dadds’ [14] review of the literature indicated there was little evidence of a reliable association between mothers’ parental attributions and treatment engagement; thus, parental attributions were an unlikely target for affecting mothers’ treatment engagement in BPT to optimise outcomes. Sawrikar and Dadds [14] suggested that a more relevant inquiry was whether problematic parental attributions hinder improvement processes within the standard BPT program, which is regarded as the most reliable path to instituting child behaviour improvements [22].

To this end, research studying family attributions in adult models of treatment relapse may help to identify the putative mediators to test this question. These models propose that families’ negative causal explanations for illness and subsequent expressed emotions of criticism, hostility, and emotional over-involvement towards the patient contribute to negative family-patient relationships that increase the risk for poor treatment outcomes [26, 27]. Patient behaviours perceived by relatives to be undesirable but under the control of the patient are most likely to be targets of criticism which is expected to result in maladaptive attempts to change the patients’ behaviour [28]. This study expands on these models to examine whether mothers’ problematic parental attributions are a risk marker of enduring negative parent–child relationships that lead to ongoing use of harsh discipline and thus poor child behaviour outcomes.

Problematic parental attributions are known to impact the affective quality of the parent–child relationship which may decrease the family’s propensity to benefit from participating in BPT. Previous research shows that mothers’ internal and stable parental attributions for problem behaviours elicit negative parental feelings and expressed emotions towards the child [29–32], and that mothers’ negative expressed emotions predict poorer outcomes for children with conduct problems [33]. Further, difficulties in down regulating negative parental feelings are known to perpetuate coercive and harsh parenting behaviours [34, 35]. No research has attempted to translate the study of attribution-relational-parenting processes within BPT; however, it may be critical given that a predictor of parent training success is improvements in parent–child relationships and decreased use of harsh parenting [36, 37]. The current study provides the first mediational test that individual differences in improvements in parental feelings and harsh discipline mediate the effects of problematic parental attributions on child behaviour outcomes.

In summary, the primary aim of the mediation analyses was to test alternative accounts of how mothers’ parental attributions affect child behaviour outcomes in BPT by examining putative mechanisms conferring the risk of mothers’ problematic parental attributions during and after treatment. The current study uses data collected from a single group treatment study used to evaluate whether problematic parental attributions, measured at pre-treatment and as pre- to post-treatment changes in parental attributions, uniquely predict child outcomes [8]. As noted in Sawrikar et al. [8], changes in parent and child outcomes in this study are attributable to both treatment effects and other concurrent changes in child and family functioning occurring during the treatment period which is consistent with anticipated outcomes of BPT [38, 39].

Figure 1 outlines the serial mediation model used to test the main hypotheses. First, to examine the possible presence of mediation it was hypothesised that (i) problematic parental attributions were associated with smaller improvements in negative parental feelings at the end of treatment (path a), (ii) smaller improvements in negative parental feelings were associated with greater use of harsh discipline (path b), and (iii) greater use of harsh discipline was associated with worse child behaviour outcomes (path c). Second, it was hypothesised that individual differences in improvements in negative parental feelings mediated the association between problematic parental attributions and mothers’ use of harsh discipline (indirect path a × b) and child behaviour outcomes (indirect path a × d). Finally, it was hypothesised that the association between problematic parental attributions and child behaviour outcomes was mediated by individual differences in improvements in negative parental feelings and mothers’ use of harsh discipline (indirect path a × b × c).

**Fig. 1:**
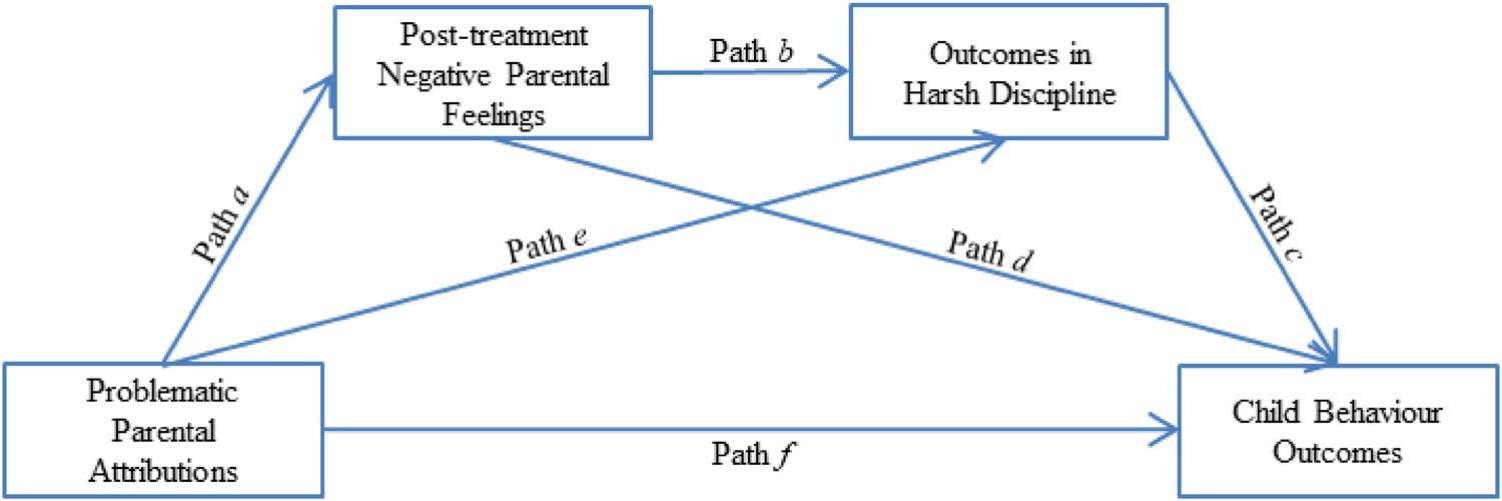
Serial mediation model testing putative mediators between problematic parental attributions and child behaviour outcomes

## Method

### Participants and Procedures

Data were collected from *N *= 169 families who completed parent training for child conduct problems at one of two Child Behaviour Research Clinic (CBRC) sites in Sydney, Australia. The two CBRC sites provide clinic-based BPT treatments to families throughout New South Wales, the most populous state of Australia. Families were eligible to participate if the referred child was aged from 3 to 16 years and had a primary presenting problem consistent with a diagnosis of Oppositional Defiant Disorder (ODD) or Conduct Disorder (CD). Children demonstrating sub-diagnostic levels of ODD or CD were also included, while children with co-occurring Attention-deficit Hyperactivity Disorder (ADHD), Anxiety Disorder, and/or Depressive Disorder were eligible for participation. Children with an intellectual disability or an Autism Spectrum Disorder (ASD) diagnosis were excluded from participation. Pre-treatment diagnostic status was determined by the Diagnostic Interview Schedule for Children, Adolescent, and Parents [40], a semi-structured diagnostic interview administered to both parents by the treating clinician. Post-treatment and 3-month follow-up diagnostic assessments were independently completed by a psychologist unfamiliar with the case. Parents also completed a battery of questionnaires at each of the different assessments. Demographic information was collected at pre-treatment, while measures of parental attributions, negative parental feelings, and use of harsh discipline were collected at the pre-treatment, post-treatment, and 3-month follow-up assessments. Parents provided informed consent prior to research participation and data were collected in accordance with ethical guidelines provided by the Human Research Ethics Committee.

Sample characteristics of participants used in the current study are summarised in Table 1. Figures relate to the *N *= 163 families where the mother had completed pre-treatment assessment questionnaires. In general, the referred child was male and from intact families where parents were either married or in a de factor living arrangement. Prior to treatment, children were rated to have conduct problems consistent with a diagnosis of either ODD or CD using DSM-IV criteria [41]. Children were generally young with 93.9% of children aged 10 years or younger. The clinical profile of participants indicated that the most common child psychopathology was comorbid conduct problems and ADHD (51.5%), conduct problems only (24.5%), comorbid conduct problems, ADHD, and mood disorder (13.5%), and comorbid conduct problems and mood disorder (10.4%). Families receiving BPT at site 2 were from a lower socio-economic background and children had greater ratings of ADHD severity to those receiving BPT at site 1.

**Table 1:** Psychosocial demographic data of families from two CBRC sites

Sample characteristics	Site 1	Site 2	Statistics
CP severity M (SD)	3.94 (.83)	4.06 (.73)	F(1,161) = .99
ADHD severity M (SD)	1.91 (1.95)	2.57 (1.73)	F(1,161) = 5.12*
Anx/Dep severity M (SD)	.61 (1.26)	.92 (1.58)	F(1,161) = 1.74
Child’s age M (SD)	7.09 (2.79)	6.77 (1.74)	F(1,161) = .81
Child’s gender N (%)			
Male	53 (40%)	80 (60%)	χ^2^ (1) = .10
Female	11 (37%)	19 (63%)	
Marital status N (%)			
Married/defacto	51 (43%)	69 (58%)	χ^2^ (1) = 2.00
Single parent	13 (30%)	30 (70%)	
Maternal depression M (SD)	4.27 (4.04)	5.68 (5.56)	F(1,161) = 3.10
SEIFA rank M (SD)	8.47 (2.17)	3.44 (1.40)	F(1,161) = 323.48*

Diagnostic severity ratings were measured using the DISCAP and grouped as non-clinical [1, 2], sub-diagnostic [3], and diagnostic [4–6]

*CP* conduct problems, *ADHD* Attention Deficit Hyperactivity Disorder, *Anx* anxiety, *Dep* depression, *N* frequency, *M* mean, *SD* standard deviation, *SEIFA* Socioeconomic Indexes for Areas [51]

*p value < .05

### Parenting Intervention

All families received the Integrated Family Intervention for Child Conduct Problems [42], a manualised social-learning based parenting intervention shown to be effective in reducing child externalising problems [43–50]. Families participated as part of a randomised control trial evaluating the efficacy of the parenting program in web-based versions that included videoconferencing with a practitioner compared to standard face–face BPT sessions. Details of the programs are provided in [50], with results indicating that the program is equally efficacious in face–face and web-based versions [see also 48, 49]. Clinical psychologists with a minimum of at least 1-year Masters Level training with specialist placements in behavioural parent management training administered the treatment.

### Measures

#### Demographic Information

Psychosocial demographic information was collected as part of the pre-treatment questionnaire battery. Socio-economic status was determined by the Socio-Economic Indexes for Areas (SEIFA) that ranks residential areas according to relative socio-economic advantage and disadvantage [51]. Marital status was recoded into single parent status where families were deemed to be single parent if parents reported to be separated, divorced, or single.

#### Diagnostic Interview Schedule for Children, Adolescents, and Parents (DISCAP)

The DISCAP [40] is a semi-structured diagnostic interview administered to parents based on the Diagnostic and Statistical Manual of Mental Disorders, 4th Edition [41]. The DISCAP provides diagnosis and rating of clinical severity. Severity ratings are scored on a 6-point scale with ratings reflecting non-clinical [1, 2], sub-diagnostic [3], and diagnostic [4–6] severity. Rater agreements (Κappa) on diagnostic category for primary and secondary diagnoses were .73 and .71 respectively. Agreement of severity levels (correlations) for primary diagnoses were: ODD/CD = .74, ADHD = .81, and Anxiety-depression = .48.

#### Parent Attribution Measure (PAM)

Child-causal attributions were measured using the Parent Attribution Measure (PAM), a 12-item self-report measure designed to assess negative causal explanations for child problem behaviour along dimensions of Intentionality, Permanence, and Disposition [18]. Respondents are asked to rate their agreement to statements on a 3-point Likert scale that ranged from 1 (‘not at all true’) to 3 (‘certainly true’). Positively worded items are reverse scored and all items are summed to represent a Total Scale measure of negative attributions for problem behaviours. The internal consistency for the Total Scale in the current study was good (α = .80). Individual difference scores reflecting pre- to post-treatment changes in parental attributions was used to measure change resistant parental attributions [8].

#### Harsh Discipline

The Corporal Punishment subscale from the Alabama Parenting Questionnaire [APQ; 52] was used to measure parental use of harsh discipline. Mothers responded to three items assessing the use of corporal punishment (e.g., “You spank your child with your hand when he/she has done something wrong”) in which items were rated on a 5-point frequency scale 1 (‘never’*)* to 5 (‘always’). The internal consistency was moderate for the Corporal Punishment scale (α = .65).

#### Parental Feelings Questionnaire (PFQ)

A seven-item version of the PFQ [53] assessed parental feelings about the child. The shortened PFQ consists of three positive items (e.g., “I feel happy about my relationship with my child”) and four negative items (e.g., “I feel frustrated by my child”), in which parents responded on a 5-point Likert scale (‘definitely true’ to ‘definitely not true’). Positive items were reversed scored and summed with negative items to create a total score for negative feelings. The short version of the PFQ was previously used to study parental affect with good reliability [54]. The internal consistency for this measure in the current study was good (α_mothers_ = .73).

### Data Analytic Plan

Mediation analyses were conducted using path analysis in MPlus (version 8) with full information maximum likelihood (FIML) estimation. Models were judged to be appropriate if it met pre-specified goodness-of-fit criteria determined by a multiple-index strategy [55] that included the model Chi square (*p* value > .05), comparative fit index (CFI; .95), and the root mean square error of approximation (RMSEA; .05). Path analytic models varied in when parental attributions (PAM Total Scale) and child behaviours outcomes (DISCAP ratings of conduct problems) were assessed: [1] mothers’ pre-treatment parental attributions on post-treatment outcomes, [2] mothers’ pre-treatment parental attributions on 3-month follow-up outcomes, and [3] pre- to post-treatment changes in mothers’ parental attributions on 3-month follow-up outcomes (i.e., measuring the association between change resistant parental attributions and outcomes).

Post-treatment ratings of negative parental feelings (PFQ score) and ratings of harsh discipline (APQ Corporal Punishment scale) at the time of assessing child behaviour outcomes were entered as mediators in serial mediation models (Fig. 1). Consistent with recent recommendations for testing mediation [56], individual components of the indirect effect pathway (‘direct effects’) were first tested for significance to examine the presence of mediating effects. The significance of mediating effects was subsequently assessed by bootstrapping with bias corrected confidence intervals for the indirect effect (10,000 resamples; Hayes 2009). A single-group design was first used to maximise analytic power, which was supported by findings that the intervention was equally efficacious across delivery modes and treatment sites [48–50]. Invariance testing using multigroup analyses examined replicability across treatment sites (group 1: site 1; group 2: site 2) and delivery modes (group 1: face–face; group 2: telehealth). Invariance was determined using the Satorra-Bentler Scaled Chi square Difference Test [57] whereby a model which constrained direct effects to be equal across groups was compared with a model which allowed direct effects to be freely estimated among groups. Indirect effects were also tested for equivalence across groups as evidence of invariance in indirect parameter estimates [58]. Significance testing was at α = .05 level.

Covariates were chosen to control for individual differences in baseline measures that could influence study outcomes. Pre-treatment scores on severity of conduct problems, severity of ADHD, negative parental feelings, mothers’ use of harsh discipline, child age, and number of sessions were added as covariate regression paths where child behaviour outcomes was the dependent variable to control for their potential influence on how parental attributions affect BPT outcomes [13, 59]. Number of sessions also controlled for differences in treatment duration which may influence the amount of change in parental attributions and parental feelings important to predicting treatment outcomes. Pre-treatment scores on parental feelings and harsh discipline were added as covariate regression paths where putative mediators were the dependent variables. Finally, models analysing the effects of changes in parental attributions included pre-treatment scores to control for pre-treatment differences on the predictor variable. Covariates were modelled to correlate with each other.

Missing data were associated with non-participation in follow-up assessments. The proportion of missing data was 6.10% for post-treatment ratings of conduct problems, 10.40% for 3-month follow-up ratings for conduct problems, 10.40% for post-treatment ratings of negative parental feelings, harsh discipline, and change in parental attributions, and 18.40% for 3-month follow-up ratings for use of harsh discipline. Missing data were missing completely at random [Little MCAR Test: χ^2^ (122, N = 163) = 131.06, *p* > .05].

## Results

### Preliminary Analysis

Descriptive statistics and correlations among variables are summarised in Supplementary Table 1. Families generally received 7 treatment sessions and a one-way repeated measures ANOVA indicated that the average ratings of conduct problems at the post-treatment and 3-month follow-up assessments were significantly lower than pre-treatment levels, *F*(2,280) = 114.85, p < .05, η^2^ = .45. Further, average ratings of mothers’ use of harsh discipline at the post-treatment and 3-month follow-up assessments were significantly lower than pre-treatment levels, *F*(2,244) = 29.92, p < .05, η^2^ = .20. A repeated measures ANOVA testing the assumption parental attributions and feelings change during treatment indicated a main effect for time with average post-treatment scores lower than pre-treatment scores [parental attributions: *F*(1,145) = 54.99, p < .05, η^2^ = .28; negative parental feelings: *F*(1,145) = 68.94, *p* < .05, η^2^ = .32].

Fit indices indicated that the two structural models accounting for the association between pre-treatment parental attributions and child behaviour outcomes measured at the post-treatment and 3-month follow-up assessments provided a good fit (model 1: χ^2^ (7)  = 11.31, p = .13, CFI = .97, RMSEA = .06; model 2: χ^2^ (7) = 10.26, p = .17, CFI = .98, RMSEA = .05). R-square statistics associated with these models showed that model 1 accounted for 31.2% of variance in negative parental feelings, 33.5% of variances in harsh discipline, and 28.2% of variance in severity of conduct problems at post-treatment. Model 2 accounted for 30.4% of variance in negative parental feelings at post-treatment, and 29.5% of variances in harsh discipline and 26.1% of variance in severity of conduct problems at 3-month follow-up. Fit indices for the model accounting for the association between pre- to- post-treatment changes in parental attributions and child behaviour outcomes measured at the 3-month follow-up assessments provided an excellent fit (model 3: χ^2^ (7) = 4.88, p = .67, CFI = 1.00, RMSEA = .00). Model 3 accounted for 48.2% of variance in negative parental feelings at post-treatment, and 30.3% of variances in harsh discipline and 29.1% of variance in severity of conduct problems at 3-month follow-up.

### Mediation Analyses of How Mothers’ Parental Attributions Affect BPT Outcomes

**Fig. 2:**
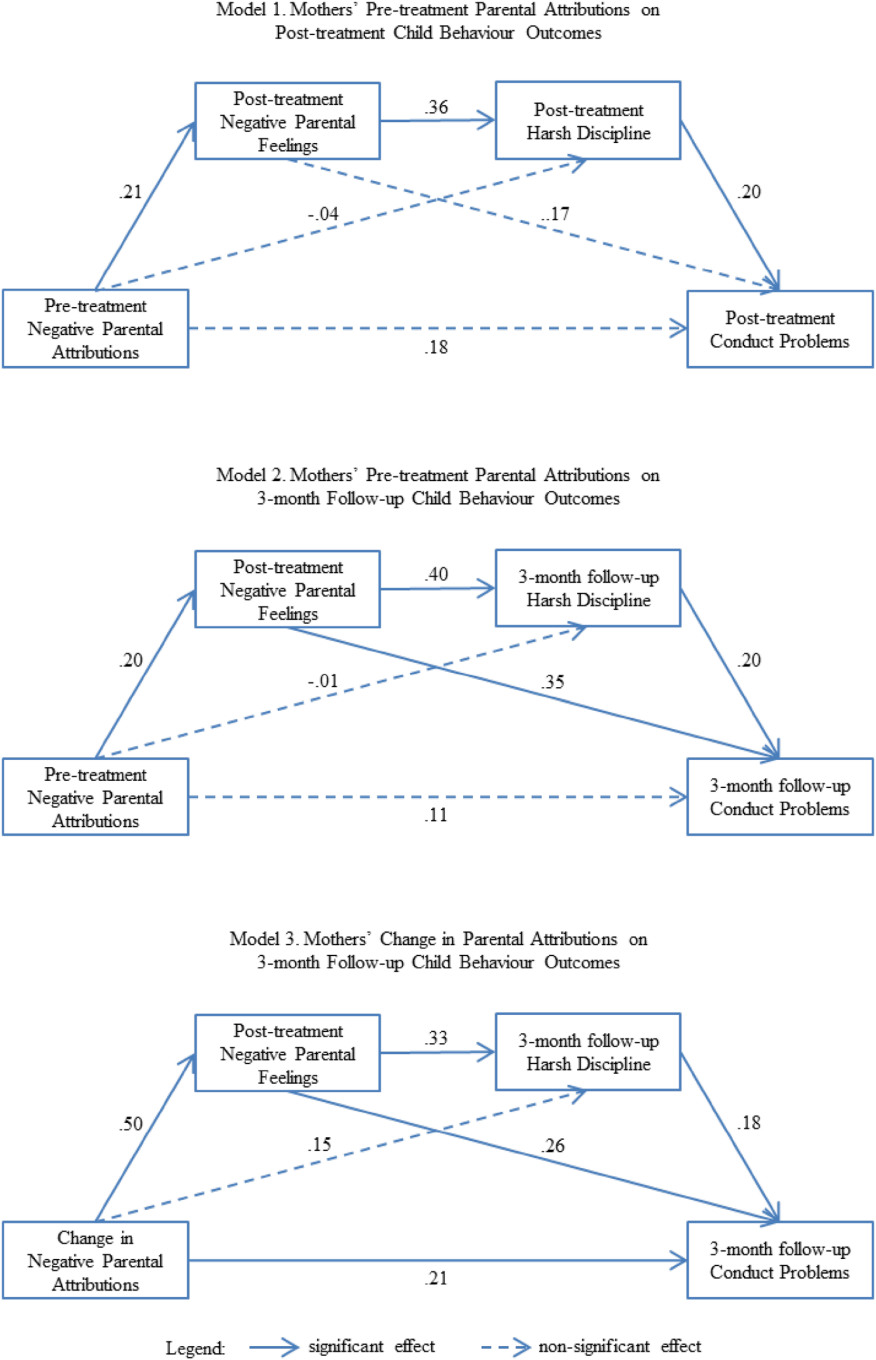
Path diagrams of mediation analyses using single-group design testing indirect effects of parental attributions on child behaviour outcomes via putative mediators. Note: path coefficients are standardised regression estimates; covariates and residuals omitted from diagram; covariates include number of treatment sessions, child age, and pre-treatment scores on negative parental attributions, negative parental feelings, harsh discipline, and severity ratings for conduct problems and ADHD

Figure 2 summarises the results for testing the significance of direct effects. In models 1 and 2, pre-treatment negative parental attributions predicted greater negative parental feelings at the end of treatment (path *a*). In turn, greater post-treatment negative parental feelings were associated with higher ratings of harsh discipline at the post-treatment and 3-month follow-up assessments (path *b*). Further, higher ratings of harsh discipline at both the post-treatment and 3-month follow-up assessments were respectively associated with higher ratings of conduct problems (path *c*). In model 3, smaller pre- to post-treatment changes in parental attributions were associated with greater post-treatment negative parental feelings (path a) which in turn predicted higher ratings of harsh discipline at the 3-month follow-up assessment (path *b*). Higher ratings of harsh discipline at the 3-month follow-up assessment were subsequently associated with higher ratings of conduct problems at the same assessment time point (path *c*). Taken together, the results indicate the possible presence of mediation along the hypothesised pathways of the mediation model.

The results for testing indirect effects associated with the putatative mediators are summarised in Fig. 1 and Table 2. In models 1 and 2, the indirect effects associated with the hypothesis that smaller improvements in parental feelings mediated the association between pre-treatment negative parental attributions and outcomes in mothers’ use of harsh discipline were significant for both the post-treatment and 3-month follow-up assessments (indirect path *a* × *b*). However, the indirect effects associated with the hypothesis that smaller improvements in parental feelings mediated the association between pre-treatment negative parental attributions and child behaviour outcomes were non-significant for both the post-treatment and 3-month follow-up assessments (indirect path *a* × *d*). Finally, the indirect effect associated with the hypothesis that the association between negative pre-treatment parental attributions and child behaviour outcomes was mediated by smaller improvements in negative parental feelings and harsh discipline were non-significant for both the post-treatment and 3-month follow-up assessments (indirect path *a* × *b *× *c*).

**Table 2:** Results from testing indirect effects via putative mediators using single-group design

Model	Indirect path	Indirect Effect (S.E)	Lower 2.5%	Upper 2.5%	p-value
1	Path *a* × *b*	.02 (.01)	.01	.05	.02*
	Path *a* × *d*	.01 (.01)	.00	.04	.22
	Path *a* × *b* × *c*	.01 (.00)	.00	.02	.10
2	Path *a* × *b*	.02 (.01)	.01	.05	.05*
	Path *a* × *d*	.02 (.01)	.00	.05	.09
	Path *a* × *b* × *c*	.01 (.00)	.00	.02	.10
3	Path *a* × *b*	.05 (.02)	.02	.10	.01*
	Path *a* × *d*	.05 (.02)	.01	.10	.03*
	Path *a* × *b* × *c*	.01 (.01)	.00	.03	.07

Model 1: predictor = pre-treatment parental attributions, outcome = post-treatment child conduct problems, mediators = post-treatment negative parental feelings and harsh discipline; Model 2: predictor = pre-treatment parental attributions, outcome = 3-month follow-up child conduct problems, mediators = post-treatment negative parental feelings and 3-month follow-up harsh discipline; Model 3: predictor = changes in parental attributions, outcome = 3-month follow-up child conduct problems, mediators = post-treatment negative parental feelings and 3-month follow-up harsh discipline; changes in parental attributions = post- minus pre-treatment parental attributions; paths defined in Fig. 1

*p-value < .05

In model 3, the indirect effect associated with the hypothesis that smaller improvements in negative parental feelings mediated the association between change resistant parental attributions and 3-month follow-up outcomes in harsh discipline was significant (indirect path *a* × *b*). Further, the indirect effect associated with the hypothesis that smaller improvements in negative parental feelings mediated the association between change resistant parental attributions and 3-month follow-up child behaviour outcomes was significant (indirect path *a* × *d*). However, the indirect effect associated with the hypothesis that the association between change resistant parental attributions and child behaviour outcomes (3-month follow-up) was mediated by smaller improvements in negative parental feelings (post-treatment) and harsh discipline (3-month follow-up) was non-significant (indirect path *a* × *b *× *c*).

Results from invariance testing indicated equivalence in direct effects across treatment sites (model 1: Satorra–Bentler Scaled χ^2^ (6)  = 2.24, *p *= .90; model 2: Satorra–Bentler Scaled χ^2^ (6) = 2.69, *p *= .85; model 3: Satorra–Bentler Scaled χ^2^ (6) = 10.15, *p *= .12) and delivery modes (model 1: Satorra–Bentler Scaled χ^2^ (6) = 6.34, *p *= .39; model 2: Satorra–Bentler Scaled χ^2^ (6) = 2.76, *p *= .84; model 3: Satorra–Bentler Scaled χ^2^ (6) = 5.80, *p *= .45). Equivalence in indirect effects among groups was also demonstrated (Table 3).

**Table 3:** Results from invariance testing of indirect effects across treatment sites (site 1 versus site 2) and delivery modes (face–face versus telehealth)

Model	Indirect effect path	Site 1 versus site 2				Face–face versus telehealth			
		b_1_–b_2_ (SE)	Lower 2.5%	Upper 2.5%	*p*-value	b_1_–b_2_ (SE)	Lower 2.5%	Upper 2.5%	*p*-value
1	Path *a* × *b*	.00 (.02)	− .04	.02	.80	.01 (.02)	− .03	.04	.78
	Path *a* × *d*	− .02 (.03)	− .09	.02	.48	.01 (.03)	− .04	.06	.85
	Path *a* × *b* × *c*	.00 (.01)	− .02	.01	.98	.01 (.01)	− .01	.02	.50
2	Path *a* × *b*	− .01 (.02)	− .07	.03	.68	− .01 (.02)	− .06	.03	.69
	Path *a* × *d*	− .03 (.03)	− .11	.02	.31	− .01 (.03)	− .07	.05	.77
	Path *a* × *b* × *c*	.00 (.01)	− .03	.01	.85	− .01 (.01)	− .03	.00	.35
3	Path *a* × *b*	.02 (.04)	− .05	.09	.53	.00 (.04)	− .08	.07	.97
	Path *a* × *d*	− .10 (.06)	− .24	.00	.09	− .04 (.05)	− .15	.05	.43
	Path *a* × *b* × *c*	.01 (.01)	− .03	.03	.63	− .01 (.02)	− .05	.01	.52

*p-value < .05

## Discussion

The aim of the mediation analyses was to account for the association between mothers’ problematic parental attributions and BPT outcomes by examining putative mechanisms between parental attributions and child behaviour outcomes. The results indicated that problematic parental attributions, operationalised as pre-treatment negative parental attributions and change-resistant parental attributions, were associated with smaller improvements in parental feelings at the end of treatment which in turn were associated with greater immediate and long-term use of harsh discipline. Greater use of harsh discipline was in turn associated with poorer child behaviour outcomes at both the post-treatment and 3-month follow-up assessments. Partial support for the mediation hypothesis was obtained with findings that smaller improvements in parental feelings mediated the association between mothers’ change resistant parental attributions and outcomes in use of harsh discipline and child conduct problems. Findings inconsistent with expectations were that putative mediators did not mediate the association between pre-treatment parental attributions and child behaviour outcomes and that smaller improvements in parental feelings and harsh discipline did not mediate the association between measures of problematic parental attributions and child behaviour outcomes in this study.

Overall, the results indicate that associations between problematic parental attributions, negative parental feelings about the child, and harsh discipline have the potential to influence child behaviour outcomes in BPT. Best to our knowledge this study represents the first examination of these relations within BPT for child conduct problems. The findings extend research showing that mothers’ problematic parental attributions are associated with negative parental feelings that affect the quality of parent–child relationships to within the BPT context [29–32]. Our findings also replicate research showing quality of parent–child relationships predict child behaviour outcomes [33, 36]. Further, the results extend previous research examining how negative parental feelings are associated with parenting behaviours implicated in maintaining child conduct problems by showing that smaller improvements in mothers’ negative parental feelings at the end of treatment are also associated with greater post-treatment use of harsh discipline [34, 35]. The current study demonstrated these factors remain related even after participation in BPT.

With regards to mediation, smaller improvements in parental feelings about the child consistently mediated the association between problematic parental attributions and outcomes in use of harsh discipline. As previously noted, these findings expand on previous research showing problematic parental attributions promote negative parental attitudes and feelings about the child which in turn are associated with maladaptive parenting behaviours [33, 35, 60]. These results suggest that including a focus on parental attributions could enhance improvements in parenting for mothers demonstrating problematic parental attributions in treatment [14, 15]. Importantly, smaller improvements in parental feelings about the child were found to mediate the association between change-resistant parental attributions and long-term child behaviours outcomes. These findings represent new findings in the literature and are significant because they clarify what role parental attributions may have in influencing BPT outcomes in the treatment of conduct problems. That is, the results suggest that a focus on problematic parental attribution could potentially optimise child behaviour outcomes by helping to address enduring negative emotional qualities of the parent–child relationship that places some families at risk for poor treatment outcomes.

Despite these significant findings, certain results from the mediation analyses warrant specific discussion. In contrast to models examining change resistant parental attributions, significant mediating effects were not found for pre-treatment parental attributions on child behaviour outcomes despite significant direct effects suggesting the possible presence of mediation. Further, the standardised coefficients suggested a small to moderate effect for pre-treatment parental attributions on post-treatment negative parental feelings compared to a large effect when change-resistant parental attributions was the predictor variable [61]. This pattern of results may suggest that effects sizes related to mediation are greater at the end of treatment where mothers’ change resistant parental attributions represents a more proximal predictor of overall child behaviour outcomes [8, 13]. Further, outcomes in harsh discipline did not mediate the association between smaller improvements in parental feelings associated with change resistant parental attributions on child behaviour outcomes. It should be noted here that there was a trend toward statistical significance for the serial indirect effect (*p* value = .07) associated with this hypothesis; thus, outcomes in harsh discipline may still have an important role in explaining how problematic parental attributions and concurrent negative parental feelings influence child behaviour outcomes. Nonetheless, given that mediation was found for negative parental feelings at the end of treatment by contrast, the pattern of results makes a prima facie case that smaller improvements in negative parental feelings provides a better account of how problematic parental attributions affect BPT outcomes.

As such, the current results suggest that negative emotional aspects of the parent–child relationship may act as a mechanism conferring the risk of problematic parental attributions in BPT. The results extend previous research identifying problematic parental attributions as a risk factor for poor outcomes [8, 12, 17] by demonstrating that they predict smaller improvements in the emotional quality of parent–child relationships which in turn predict poorer treatment outcomes. We suggest that these results are best interpreted within models of reducing relapse in adults where negative attributions and expressed emotion are hypothesised to influence treatment outcomes [26]. These models recommend that standard interventions include a focus on family attributions to help remediate processes relating negative emotional aspects of family relationships to poor outcomes. Deciding when to focus on parental attributions in treatment remain unclear, particularly given mediation was observed for measures of change-resistant parental attributions and not for pre-treatment parental attributions. However, an ‘embedded’ approach to tailoring BPT is recommended to resolve this whereby parental attributions are assessed and monitored throughout treatment to determine if they are blocking treatment implementation [14]. Attribution theory then becomes part of a repertoire of tools for practitioners when problematic parental attributions limit improvements in parent–child relationships that help achieve positive child behaviour outcomes [7].

We note that the current study did not assess which clinical techniques to use while working with negative parental attributions. Previous researchers advocating for an embedded approach have suggested the use of classical cognitive restructuring/disputation techniques [7]. However, such techniques are yet to demonstrate efficacy in enhancing outcomes when used as a BPT module [22]. Alternatively, defusing or distancing the parent from maladaptive parental attributions as outlined within acceptance and commitment therapy and mindfulness-based interventions may be appropriate [62, 63]. Parental attributions may also not represent the primary focus of adjunctive components [64]. Rather, attention could be paid to directly improving the affective quality of parent–child relationships to minimise the impact of negative parental attributions on outcomes. A research agenda comparing techniques would help elucidate clinical approaches suitable for addressing parental attributions in BPT.

Other limitations of the current study present as possible directions for future research. We examined improvements in parental attributions and parent–child relationships as related outcomes consistent with research studying secondary outcomes of BPT [13, 38, 39]. However, changes in parental attributions and outcomes in parental feelings about the child were assessed at the same time; thus, the results do not tease out any putative directionality of influence. Mediation models also did not include variables evaluating how pre-treatment or treatment-related changes in child behaviours affect outcomes. We chose to model parent variables in BPT for parsimony based on an a priori theoretical model [26] and to maximise analytic power; thus, the results cannot rule out possible interactional effects between child and parent variables. It should be noted, however, that the model in the current study relates to a follow-up analysis that previously showed that problematic parental attributions uniquely predict child outcomes after controlling for changes in child behaviour during treatment [8]. Future research will ideally involve frequent assessments of parental attributions, parenting, and child functioning within a longitudinal framework to reliably examine change processes within treatment. Finally, future use of observational measures like expressed emotion will help test the replicability of results when using alternative methods of assessment that minimise shared method variance. Observational methods are also useful in proposing specific behaviours as modifiable targets which could potentially limit the impact of problematic parental attributions on outcomes [65].

In sum, the current study represents the first attempt to study how parental attributions affect treatment outcomes in BPT using mediation analysis. The analyses revealed that a potential mechanism of how parental attributions affect outcomes includes negative parental feelings about the child. These findings bolster the general case that a focus on parental attributions has the potential to optimise parent training outcomes in the treatment of child conduct problems by facilitating improvements in parent–child relationships. The problem is that the field remains relatively silent on evidence-based clinical techniques to achieve this. In conclusion therefore we suggest that the field would benefit from practitioners and program developers thinking about how to build personalised interventions targeting parental attributions that can be manualised for clinical replication and research evaluation.

## Summary

Parental attributions are known to predict child outcomes from participation in parent training suggesting that best possible programs might include a focus on parental attributions to optimise outcomes. The current study examined how mothers’ problematic parental attributions affect child outcomes from a positive parenting program to better understand the role of parental attributions in BPT for conduct problems. It was hypothesised that smaller improvements in parental feelings about the child and ongoing use of harsh discipline mediates the effects of problematic parental attributions on child outcomes. The results suggested that a potential mechanism of how mothers’ parental attributions affect outcomes includes negative parental feelings about the child. The results support tailoring parenting interventions to focus on parental attributions to help remediate processes relating negative emotional aspects of parent–child relationships to poor treatment outcomes.

## Electronic supplementary material

Below is the link to the electronic supplementary material.

Supplementary material 1 (DOCX 27 kb)
