# Supplementary material for: How Do Mothers’ Parental Attributions Affect Child Outcomes from a Positive Parenting Intervention? A Mediation Study
Source: Child Psychiatry Hum Dev. 2019 Nov 15;51(4):597–608. doi: 10.1007/s10578-019-00942-0 (PMC7347688; doi:10.1007/s10578-019-00942-0)
Supplement: Supplementary file 1 — Supplementary material 1 (DOCX 27 kb) [file 10578_2019_942_MOESM1_ESM.docx]

Supplementary Table 1.

*Descriptive statistics and correlations of variables in mediation models.*

|  | 1 | 2 | 3 | 4 | 5 | 6 | 7 | 8 | 9 | 10 | 11 | 12 |
| --- | --- | --- | --- | --- | --- | --- | --- | --- | --- | --- | --- | --- |
| 1. Pre-treatment CP severity | - |  |  |  |  |  |  |  |  |  |  |  |
| 2. Post-treatment CP severity | .42* | - |  |  |  |  |  |  |  |  |  |  |
| 3. 3-month follow-up severity | .18* | .43* | - |  |  |  |  |  |  |  |  |  |
| 4. Pre-treatment ADHD severity | .28* | .30* | .26* | - |  |  |  |  |  |  |  |  |
| 5. Pre-treatment parental attributions | .36* | .34* | .20* | .15 | - |  |  |  |  |  |  |  |
| 6. Changes in parental attributions^a^ | .26 | .46* | .38* | .25 | -.52* | - |  |  |  |  |  |  |
| 7. Number of sessions | -.06 | .01 | -.17* | .02 | -.11 | .02 | - |  |  |  |  |  |
| 8. Pre-treatment negative parental feelings | .24* | .20* | .11 | .10 | -.35* | -.34* | .09 | - |  |  |  |  |
| 9. Post-treatment negative parental feelings | .30* | .37* | .42* | .26* | .32* | .11 | .06 | .53* | - |  |  |  |
| 10. Pre-treatment harsh discipline | .14 | .13 | .06 | .05 | -.39* | -.20* | .08 | .30* | .20* | - |  |  |
| 11. Post-treatment harsh discipline | .20* | .27* | .20* | .06 | .19 | .13 | -.03 | .11 | .33* | .48* | - |  |
| 12. 3-month follow-up harsh discipline | .22* | .20* | .31* | .08 | .27* | .12 | -.02 | .07 | .34* | .41* | .75* | - |
| Mean | 4.01 | 2.32 | 2.40 | 2.31 | 8.58 | -2.28 | 7.10 | 21.65 | 18.26 | 4.78 | 3.82 | 3.80 |
| SD | .77 | 1.58 | 1.40 | 1.84 | 4.23 | 3.71 | 2.24 | 4.37 | 5.10 | 1.69 | 1.21 | 1.14 |
| Minimum | 3 | 0 | 0 | 0 | 1 | -19 | 3 | 7 | 7 | 3 | 3 | 3 |
| Maximum | 6 | 5 | 5 | 5 | 21 | 7 | 13 | 35 | 31 | 15 | 9 | 9 |

*Note*: changes in parental attributions = post- minus- pre-treatment parental attributions; ^a^ partial correlations reported after controlling for pre-treatment parental attributions; CP = conduct problems; ; ADHD = Attention Deficit Hyperactivity Disorder; **p*-value < .05.
